# The value of metabolic parameters on dynamic ^18^F-FDG PET/CT for predicting lymph node metastasis in non-small cell lung cancer

**DOI:** 10.3389/fonc.2026.1752947

**Published:** 2026-02-12

**Authors:** Linna Guo, Xieraili Wumener, Fen Du, Zhiheng Yao, Xinyu Yang, Shengyun Huang, Tao Sun, Ying Liang

**Affiliations:** 1 Department of Nuclear Medicine, National Cancer Center/National Clinical Research Center for Cancer/Cancer Hospital & Shenzhen Hospital, Chinese Academy of Medical Sciences and Peking Union Medical College, Shenzhen, China; 2 Lauterbur Research Center for Biomedical Imaging, Shenzhen Institute of Advanced Technology, Chinese Academy of Sciences, Shenzhen, China

**Keywords:** ^18^F-FDG, dynamic, lymph node, non-small cell lung cancer, PET/CT

## Abstract

**Objectives:**

To evaluate the predictive value of dynamic ^18^F-FDG PET/CT metabolic parameters of the primary tumor for mediastinal lymph node metastasis (LNM) in non-small cell lung cancer (NSCLC).

**Methods:**

A total of 316 patients with clinically suspected but untreated lung lesions who underwent dynamic PET/CT and static PET/CT scans from May 2021 to November 2024 were retrospectively collected in this study. Quantitative parameters including K1, k2, k3, and Ki of each lesion, were obtained by applying the irreversible two-tissue compartmental modeling using an in-house Matlab software. Time-activity curves (TACs) at the primary tumor were extracted from each dynamic ^18^F-FDG PET/CT scan. The TAC signal was then decomposed into metabolism and blood flow components through kinetic modeling. Dynamic features including area under the curve (AUC), time-to-peak (t_peak_), and slopes were then extracted from each component. Predictive analyses were performed using multivariate logistic regression to determine the predictive factors for LNM. Receiver-operating characteristic (ROC) analysis was performed to evaluate the predictive performance of models.

**Results:**

One hundred and fifteen patients who obtained LN biopsy within one month were enrolled in this study. Based on the results of the pathology, the patients were divided into LNM and non-LNM groups. The multivariate logistic regression analyses showed that the TLG, slope_10-30min_, and CA125 were independent predictive factors for LNM (*P* < 0.05, respectively). For the model comparison, composite model achieved the highest diagnostic efficacy (AUC of 0.867, sensitivity 75.5%, specificity 84.5%, accuracy 80.2%) compared with PET/CT model (AUC of 0.822, sensitivity 80.0%, specificity 72.4%, accuracy 75.7%) and clinical model (AUC of 0.792, sensitivity 49.1%, specificity 96.7%, accuracy 73.9%).

**Conclusion:**

The metabolic parameters based on dynamic and static ^18^F-FDG PET/CT combined with CA125 can improve N-staging accuracy in patients with NSCLC.

## 1 Introduction

Lung cancer is the leading cause of cancer-related deaths in both males and females worldwide. Non-small cell lung cancer (NSCLC) accounts for 85% of all lung cancers, with a high incidence rate and mortality (1). The primary route of metastasis for lung cancer is through lymph node metastasis (LNM). Research has shown that the 5-year survival rate for patients with stage N1 to N3 NSCLC was 9% to 37% (2), and surgery remains the best choice for early-stage patients. Standardized lymph node dissection was significant for patients (3). Therefore, accurate N staging of NSCLC was crucial for determining the optimal treatment (4).

Staging of mediastinal LN by endobronchial ultrasound-guided transbronchial needle aspiration (EBUS-TBNA)/mediastinoscopy had been reported to be very useful and was recommended when LNM was suspected by imaging (5). However, some LNs were difficult to evaluate using EBUS-TBNA. In addition, there would be complications such as bleeding and mediastinitis (6).

The non-invasive ^18^F-fluorodeoxyglucose (FDG) positron emission tomography/CT (PET/CT) played an important role in the differential diagnosis, staging, response assessment, and prognosis of lung cancer. In clinical practice, ^18^F-FDG PET/CT was widely used for clinical LN staging. Previous studies had shown that semi-quantitative metabolic parameters such as standardized uptake value (SUV), metabolic tumor volume (MTV), and total lesion glycolysis (TLG) were associated with tumor heterogeneity and could predict tumor invasiveness (7), LN status (8, 9), and prognosis (10, 11). However, SUVs are affected by a variety of factors such as body composition, changes in intake over time, and blood glucose level, etc (12, 13). Therefore, semi-quantitative analysis of static PET/CT (sPET/CT) images may sometimes be unreliable.

Dynamic PET/CT (dPET/CT) can generate quantitative parameter images through the compartment model, such as the Patlak-derived net inflow rate Ki, which can better reflect the true metabolic rate of the tumor than the SUV. Karakatsanis et al. established the methodological basis of dPET parametric imaging which laid a key theoretical and practical framework for subsequent whole-body dynamic PET research (14, 15). Based on this methodology, dPET has become a powerful tool for studying tumor heterogeneity and systemic diseases. Previous studies have demonstrated that the dynamic metabolic parameters of ^18^F-FDG PET/CT better reveal the pathophysiological mechanisms of the disease, and Ki was superior to SUVmax in the differential diagnosis of pulmonary nodules and lymph nodes (16–18). Moreover, the recent study reported that the time-activity curve (TAC) of dPET/CT can distinguish lung cancer and benign lung lesions (19).

However, there have been no reports on the prediction of LNM using dynamic metabolic parameters of primary tumors. Therefore, this study aims to explore the predictive value of primary tumor metabolic parameters from dPET/CT and sPET/CT imaging for LNM.

## 2 Materials and methods

### 2.1 Patients

The study was approved by the ethics committee of Cancer Hospital & Shenzhen Hospital, Chinese Academy of Medical Sciences (KYLH2022-1). All patients signed a written informed consent according to the Declaration of Helsinki before the FDG PET/CT imaging.

**Figure 1:**
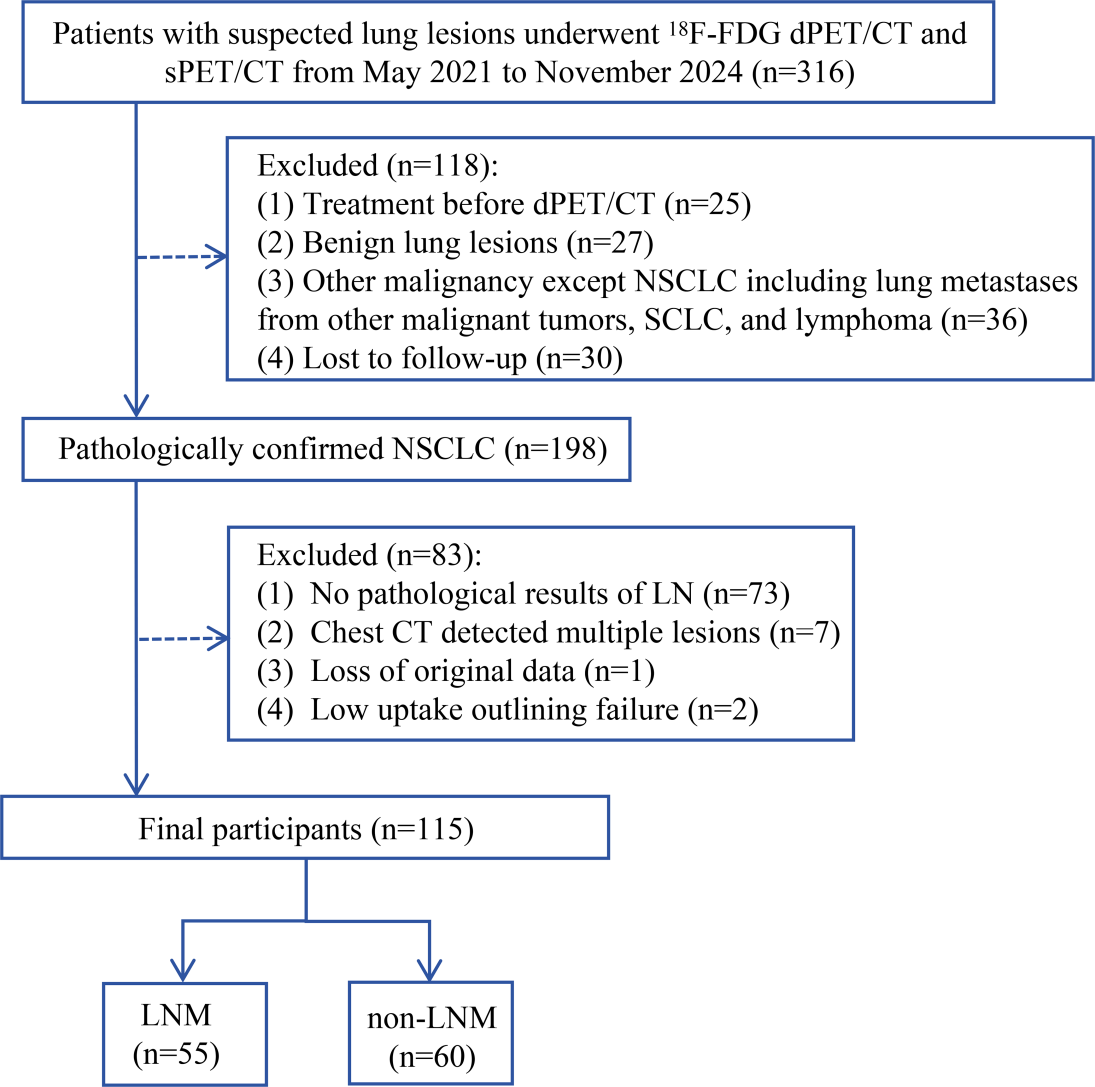
Flow chart showing the participants’ selection process for the study. LNM, lymph node metastasis.

A total of 316 patients with clinically suspected but untreated lung lesions who underwent dPET/CT (65 min, chest) and sPET/CT (10–20 min, whole body) scans from May 2021 to November 2024 were retrospectively collected in this study. Inclusion criteria were: (1) successfully underwent chest dPET/CT, breath-hold chest CT, and whole-body sPET/CT scans; (2) diagnosed with NSCLC through pathology, including surgery and EBUS biopsy; (3) not receiving anti-tumor treatment before PET/CT scan. Exclusion criteria: (1) no pathological results of mediastinal LN; (2) chest CT detected multiple nodules or masses in both lungs (at least 2 lesions); (3) the primary lesion of the lung is a pure ground glass nodule; (4) not willing to cooperate, incomplete clinical data. Finally, 115 patients were included in this study. Collect clinical characteristics of patients, including gender, age, smoking history, long diameter of primary lung lesions, pathological type, and serum tumor markers (including CEA, CYFRA21-1, CA125, SCC, NSE, and ProGRP). The flow chart of enrollment is shown in **Figure 1**.

### 2.2 PET/CT data acquisition and reconstruction

According to the previous research method (16), all the patients avoided strenuous exercise for 24 h before the PET/CT scan and fasted for at least 6 h before the scan. The scanner model was a Discovery MI PET/CT (GE Healthcare, Milwaukee, USA). The patient’s blood glucose levels were below 11.1 mmol/L. The scan protocol was as follows: First, the patients underwent a breath-hold CT of the chest with a tube voltage of 120 kV, tube current 10–220 mA, pitch 1.375:1, and a noise index of 20. The chest region, covering an axial field-of-view of 20 cm, was then scanned immediately after ^18^F-FDG injection through an intravenous indwelling needle. Dynamic list-mode PET acquisition was performed immediately after the injection.

The total duration of the scan was 65 min, with the data divided and reconstructed into 27 frames (6×10 s, 4×30 s, 4×60 s, 4×120 s, and 9×300 s). Each frame was reconstructed to a matrix of 256×256×71 voxels using the block sequential regularized expectation maximization algorithm (25 iterations and 2 subsets, post-smoothing). Attenuation and scatter corrections were performed during the reconstruction using CT-based attenuation maps. All the resulting dynamic frames were then converted to the SUV by normalizing the measured counts to the individual’s dose and weight.

After the dynamic scan, the patients underwent a whole-body CT scan from the head to the mid-femur in a supine position with the arms raised. An additional whole-body sPET scan was then performed. For both PET scans, the attenuation corrections were performed using CT data, and the PET reconstructions were performed using the Block sequential regularized expectation maximization (BSREM) reconstruction algorithm with 25 iterations and 2 subsets (16).

### 2.3 PET/CT data analysis

According to the previous research method (16), the dynamic parameters of K1, K2, K3, and Ki were obtained based on the two-tissue irreversible compartment model. The image-derived input function (IDIF) was extracted from the ascending aorta by drawing a 10-mm-diameter ROI on six consecutive slices in an image obtained by combining early time frames (0–60 s), where the effects of motion and partial volume were less prominent than in the left ventricle. The uptake difference in blood and plasma was not accounted for in this study. The 3D volume of interest (VOI) of each lesion was delineated using the semiautomatic methods with a threshold of 40% SUVmax in ITK-SNAP software (version 3.8). For the lesions with surrounding physiological uptake, 3D VOI was manually delineated slice-by-slice by 2 experienced nuclear medicine physicians. Then the segmented VOI was applied to the K1, K2, K3, and Ki parametric images to extract the quantitative measurements of each scan (16). Using a 40% threshold, the tumor burden was calculated by plotting a three-dimensional VOI based on the volume of metabolic tumor-related activity. SUVs include SUVmax, SUVpeak, SUVmean, MTV, and TLG for primary lung cancer lesions. Simultaneously analyze the length, maximum CT value (HUmax), minimum CT value (HUmin), and mean CT value (HUmean) of the primary lesion of lung cancer on breath-holding CT.

Derived from the TAC computed for the ROI, several 5 features were extracted, delineated as follows:

(1)The slope of the 10–30 minutes curve represents the trend of the curve after peaking in the first thirty minutes, reflecting changes in tissue uptake of FDG.


$$Slope_{10-30}=\frac{TAC(10)-TAC30}{interval-time}$$

(2)The time to peak reflects the time it takes for FDG to reach its maximum concentration in tissues.


$$t_{peak}=\arg\max_{t}f(t)$$

(3)The peak of the curve responds to the maximum uptake of FDG by the target tissue over some time.


$$TAC_{\max}=f\left(t_{peak}\right)$$

(4)The area under the curve reflects the total FDG exposure of the target organization during the period.


$$AUC=\sum_{i=1}^{n-1}\frac{c(t_{i})+C(t_{i+1})}{2}\times (t_{i+1}-t_{i})$$

(5)The slope of the curve fit from the starting point to the peak, this slope indicates the rate at which FDG rapidly accumulates into the tissue during the initial period, reflecting the metabolic activity of the corresponding region.


$$Slope_{0-\max}=\frac{TAC(max)-TAC(0)}{interval-time}$$

### 2.4 Statistical analyses

Statistical analysis was conducted using SPSS 25.0 software. The difference between groups was compared using two independent t-test, Mann-Whitney U test, or chi-square test. A multivariate logistic regression analysis was used to establish the predictive model formula. Receiver operating characteristics (ROC) curves were used to evaluate the predictive efficacy of different models. Use MedCalc 20.0 software to draw ROC curves, evaluate the predictive performance of different models, perform parallel Delong tests, and compare the differences in area under the curve (AUC) between different models. *P* < 0.05 was considered statistically significant.

## 3 Result

### 3.1 General characteristics

A total of 115 patients with newly diagnosed NSCLC were enrolled in this study, including 67 males (58.3%) and 48 females (41.7%) with an age of 61.6 ± 8.5 years (range, 30-85y). Patients and lesion characteristics are presented in **Table 1**. Among them, 69 patients underwent lobectomy and systematic lymph node dissection, 1 patient underwent lung segment and lymph node sampling, and 45 patients underwent EBUS lymph node biopsy. LNM occurred in 55 patients (47.8%). Of the 55 patients with LNM, 34 were confirmed by EBUS and 21 were confirmed by surgery. Among 60 patients with non-LNM, 11 cases were confirmed by EBUS and 49 cases were confirmed by surgery. Because of the limited number of cases in this study, 11 patients with non-LNM confirmed by EBUS were not excluded. Histopathological examination revealed 17 squamous cell carcinomas, 89 adenocarcinomas, and 9 carcinomas of other including 3 adenosquamous carcinomas, 1 carcinoid tumor, 2 large cell neuroendocrine carcinoma, 2 lymphoepithelial carcinomas of the lung, and 1 pleomorphic carcinoma.

**Table 1:** Characteristics of the patients and primary lung cancer lesions.

Characteristics	N (%)
Age, years	61.6 ± 8.5
Sex	
Male	67 (58.3%)
Female	48 (41.7%)
Smoking history	
Yes	32 (27.8%)
No	83 (72.2%)
Lobe	
RUL	28 (24.3%)
RML	24 (20.9%)
RLL	16 (13.9%)
RMLL	1 (0.9%)
LUL	23 (20.0%)
LLL	23 (20.0%)
Histological type	
Squamous cell carcinoma	17 (14.8%)
Adenocarcinoma	89 (77.4%)
Others^a^	9 (7.8%)
N stage	
N0	60 (52.2%)
N1	10 (8.7%)
N2	27 (23.5%)
N3	18 (15.7%)

RUL, right upper lobe; RM, right middle lobe; RLL, right lower lobe; LUL, left upper lobe; LLL, left lower lobe; RMLL, right middle and lower lobe. ^a^: 3 adenosquamous carcinomas, 1 carcinoid tumor, 2 large cell neuroendocrine carcinoma, 2 lymphoepithelial carcinomas of the lung, and 1 pleomorphic carcinoma.

### 3.2 PET/CT parameters and clinical characteristics analysis of LNM and non-LNM

**Table 2** shows the parameters analysis of primary tumor in dPET/CT and sPET/CT scans in the LNM group and non-LNM group. The PET/CT parameters of the LNM group, including primary lesion length, CT density, SUVmax, SUVmean, SUVpeak, MTV, TLG, Ki, Slope_10-30_, time_tacmax_, and AUC were all higher than those of the non-LNM group, and such differences were statistically significant (*P* < 0.05, respectively). The dynamic parameter Slope_0-max_ of the LNM group was lower than that of the non-LNM group (*P* < 0.05). However, K1, K2, K3, and TAC_max_ could not well differentiate LNM and non-LNM (all *P*>0.05). Besides, serum tumor markers such as CEA, CA125, and Cyfra21–1 of the LNM group were all higher than those of the non-LNM group (all *P* < 0.05).

**Table 2:** PET/CT parameters and clinical characteristics analysis.

parameter	non-LNM (n=60)	LNM (n=55)	*P* value
Age, years	62.23 ± 9.90	57.89 ± 10.91	0.027^*^
Sex			0.459
male	33	34	
female	27	21	
Smoking history			0.619
Yes	17	15	
No	43	40	
HUmax (HU)	55.00(45.75, 69.25)	64.00(52.00, 73.50)	0.009^*^
HUmin (HU)	-22.50(-247.25, -3.75)	-5.00(-18.00, 3.00)	0.001^*^
HUmean (HU)	23.65(-19.76, 32.48)	31.10(24.14, 37.21)	0.000^**^
SUVmax	10.78 ± 6.90	14.17 ± 6.25	0.007^*^
SUVmean	6.41 ± 4.10	8.47 ± 3.83	0.007^*^
SUVpeak	7.90 ± 5.53	10.29 ± 4.92	0.017^*^
MTV	4.60(1.98, 10.58)	7.80(3.70, 18.90)	0.044^*^
TLG	22.15(6.03, 68.65)	57.50(26.25, 184.90)	0.003^*^
Long-diameter (cm)	2.70(2.10, 3.83)	3.60(2.45, 4.55)	0.022^*^
K1 (ml/g/min)	0.124(0.086, 0.209)	0.124(0.087, 0.168)	0.886
K2 (min^-1^)	0.293(0.164, 0.455)	0.183(0.092, 0.301)	0.051
K3 (min^-1^)	0.063 ± 0.0412	0.071 ± 0.037	0.281
Ki (ml/g/min)	0.022(0.011, 0.032)	0.037(0.025, 0.049)	0.000^**^
Slope_10−30_	0.1133 ± 0.1350	0.2252 ± 0.1683	0.000^**^
time_tacmax_	0.583(0.417, 0.323)	62.500(52.500, 62.500)	0.023^*^
TAC_max_	17.925(13.470, 22.445)	21.220(14.495, 27.910)	0.122
AUC	590.480(403.316, 865.447)	874.955(591.114, 1152.868)	0.002^*^
Slope_0-max_	25.162(0.320, 51.290)	0.403(0.283, 0.711)	0.015^*^
CEA (ng/ml)	2.590(1.755, 4.738)	4.690(2.385, 14.405)	0.001^*^
ProGRP (pg/ml)	43.405(37.542, 48.582)	40.600(30.650, 53.365)	0.401
Cyfra21-1 (ng/ml)	2.760(1.993, 3.948)	4.49(2.600, 7.510)	0.002^*^
CA125 (U/ml)	10.250(8.500, 15.325)	27.600(11.250, 73.450)	0.000^**^
NSE (ng/ml)	12.090(10.050, 14.395)	13.060(10.995, 14.975)	0.077
SCC (ng/ml)	1.020(0.708, 1.380)	1.020(0.760, 1.685)	0.580

SUVmax, maximum standardized uptake value; SUVmean, mean standardized uptake value; SUVpeak, peak standardized uptake value; MTV, metabolic tumor volume; TLG, total lesion glycolysis; TAC, time-activity curve; AUC, area under the curve; CEA, carcinoembryonic antigen; ProGRP, pro-gastrin-releasing peptide; Cyfra21-1, cytokeratin 19 fragment; CA125, carbohydrate antigen 125; NSE, neuron-specific enolase; SCC, squamous cell carcinoma antigen. Statistical significance: ^*^P < 0.05; ^**^P < 0.001; the same below.

### 3.3 Predictive factor analysis and prediction model of mediastinal LNM

Incorporate parameters with statistically significant differences into the multivariate logistic regression to obtain a PET/CT prediction model for LNM. Score_PET/CT_=-1.953 + 0.009×HUmax+0.001×HUmin+0.027×HUmean+0.040×SUVmax+0.067×MTV-0.010×TLG+14.302×Ki+10.296×Slope_10-30_+0.002×time_tacmax_-0.002×AUC-0.003×Slope_0-max_.

The Hosmer-Lemeshow test showed that the PET/CT model fitted well (R^2^ = 0.416, P = 0.864). It can be seen that both TLG and Slope_10−30_ are independent predictive factors for LNM in NSCLC among the parameters of the model (**Table 3**). In the clinical model, CA125 is an independent predictor of LNM in NSCLC (**Table 4**). Score _clinical model_ =-1.668 + 0.049×primary lesion length + 0.088×CEA + 0.003×Cyfra21-1 + 0.031×CA125. Combined with clinical parameter composite model: Score_composite model_ =-4.04 + 4.164 × Score_PET/CT_ + 4.216 × Score_clinical model_.

**Table 3:** Multivariate logistic regression analysis of PET/CT parameters for LNM.

PET/CT parameters	*b* value	OR (95%*CI*)	*P* value
SUVmax	0.040	1.040 (0.899~1.205)	0.597
MTV	0.067	1.070 (1.000~1.144)	0.051
TLG	-0.010	1.099 (0.981~1.198)	0.013^*^
Ki (ml/g/min)	14.302	1.154 (0.910~1.463)	0.238
Slope_10−30_	10.296	1.108 (1.015~1.211)	0.022^*^
time_tacmax_	0.002	1.002 (0.975~1.030)	0.889
AUC	-0.002	0.998 (0.995~1.001)	0.172
Slope_0-max_	-0.003	0.997 (0.976~1.018)	0.762
HUmax (HU)	0.009	1.009 (0.965~1.055)	0.687
HUmin (HU)	0.001	1.001 (0.995~1.006)	0.215
HUmean (HU)	0.027	1.027 (0.985~1.072)	0.162

**Table 4:** Multivariate logistic regression analysis of Clinical characteristics for LNM.

Clinical parameter	*b* value	OR (95%*CI*)	*P* value
Long-diameter (cm)	0.049	1.050 (0.758~1.455)	0.769
CEA (ng/ml)	0.088	1.092 (0.991~1.203)	0.076
Cyfra21-1 (ng/ml)	0.003	1.003 (0.873~1.151)	0.970
CA125 (U/ml)	0.031	1.032 (1.010~1.054)	0.005^*^

### 3.4 ROC curve analysis and predictive performance of multiple models

**Figure 2:**
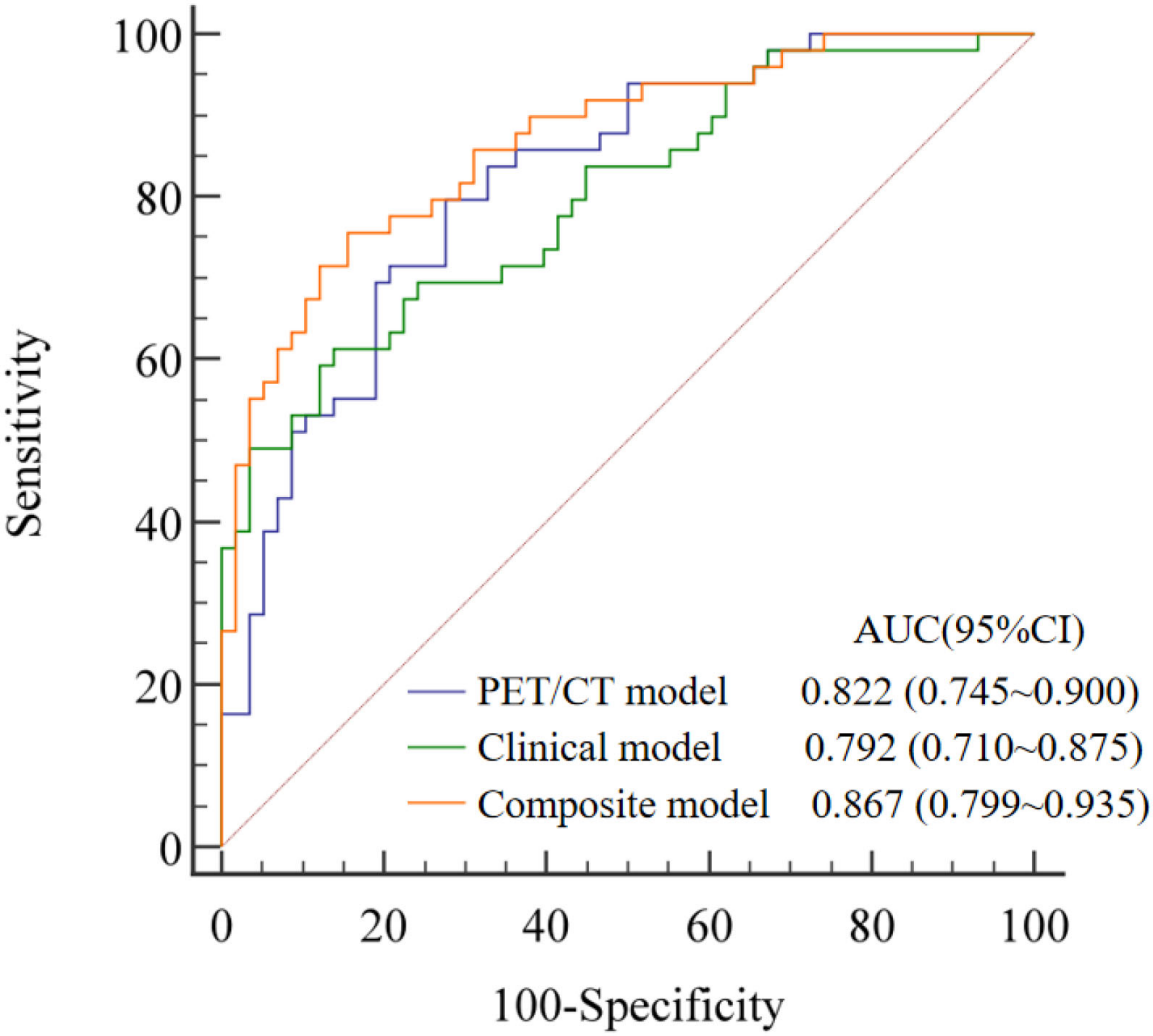
The ROC curves of three models for predicting LNM in 115 NSCLC patients.

**Figure 3:**
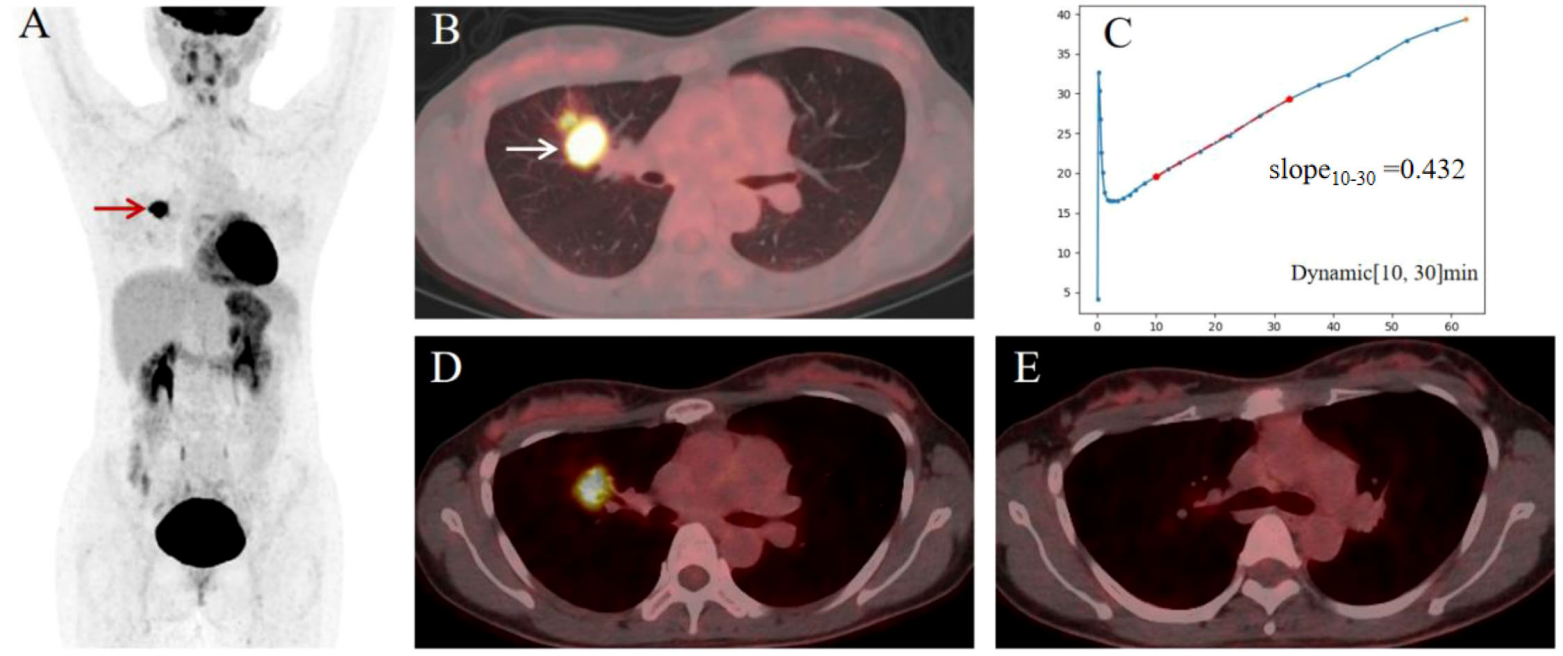
A 35-year-old female patient with newly diagnosed lung adenocarcinoma underwent dynamic and static PET/CT imaging for pre-treatment staging. **(A)** The maximum intensity projection image (MIP) of static ^18^F-FDG PET/CT. **(B)** Static PET/CT showed an FDG-avid solid nodules (2.6 × 2.0cm) in the upper lobe of the right lung (white arrow, SUVmax of 15.2, MTV of 4.7, TLG of 44.2). **(D, E)** showed no suspicious metastatic lymph nodes. **(C)** Visualization of the feature Slope_10–30_ within the TAC of dynamic PET. Right upper lobectomy and systematic lymph node dissection were performed within 1 week after PET/CT examination. Further surgical pathology identified lung adenocarcinoma with LNM, pT3N1.

The AUCs of PET/CT model, clinical model, and composite model for predicting NSCLC LNM were 0.822 (95% *CI*: 0.745~0.900, *P* < 0.001), 0.792 (95% *CI*: 0.710~0.875, *P* < 0.001), and 0.867 (95% *CI*: 0.799~0.935, *P* < 0.001), respectively (**Figure 2**). The AUC of the composite model was higher than that of the PET/CT model and clinical model, and the difference were statistically significant (*P* value: 0.033, 0.049). The difference between the AUC of the PET/CT model and the clinical model was not statistically significant (*P* = 0.680). The sensitivity, specificity, and accuracy of the three models in predicting LNM were 80.0%, 72.4%, 75.7% for the PET/CT model, and 49.1%, 96.7%, 73.9% for the clinical model, and 75.5%, 84.5%, 80.2% for the composite model, respectively. A typical case was presented in **Figure 3**.

## 4 Discussion

Accurate N staging is the key to developing treatment plans for NSCLC patients. In this study, we investigated the predictive value of dPET/CT and sPET/CT metabolic parameters and serum tumor markers for LNM in NSCLC primary tumors and established predictive models. Our findings demonstrate that TLG, slope_10-30min_, and CA125 were independent predictive features for LNM (*P* < 0.05, respectively). For the model comparison, the composite model achieved the highest diagnostic efficacy compared with the PET/CT model and clinical model, which provides a potential incremental value for accurate N staging of patients with NSCLC.

Previous studies have reported that SUVmax and SUVpeak are considered important and independent predictive features for NSCLC LNM, indicating that the higher SUV of the primary tumor, the more likely LNM is to occur (20, 21). In our results, although SUVs were higher in the LNM group than in the non-LNM group, and the difference was statistically significant, it was not an independent predictor of LNM. The correlation between SUVmax and tumor proliferative activity has been widely confirmed, but its limitation was that it only represents a single time point metabolic peak (22). MTV and TLG were comprehensive parameters that integrate tumor metabolic activity and tumor volume, which can more accurately reflect the biological behavior, prognosis, and post-treatment response of tumors (7, 8). Wang et al. and Park et al. showed that MTV was an independent predictor of LNM (23, 24). On the contrary, our results indicated that TLG was an independent risk factor for LNM. It may be because their participants were T1–2 NSCLC, and the study predicted occult LNM, while our study included N3 patients. Therefore, the predictive value of MTV and TLG needs to be further confirmed by large sample and multi-center clinical studies in the future.

DPET/CT has been developed to obtain absolute quantitative metabolic parameters (such as K1, K2, K3, and Ki) by continuously obtaining imaging data for a certain period. Compared with the traditional static semi-quantitative metabolic parameters SUV, these absolute quantitative metabolic parameters have potential advantages in reflecting tumor characteristics and differential diagnosis of benign and malignant tumors (25, 26). A previous study in our institution has confirmed that Ki was an effective indicator for differentiating benign and malignant mediastinal and hilar LNs in patients with lung cancer (14). The mediastinal and hilar LNs were susceptible to interference from adjacent lymph nodes when sketching VOI, which may affect the reliability of metabolic parameter results. The lung lesions were less affected by the adjacent tissues. Therefore, the state of LN can be predicted by the dynamic parameters of the primary tumor. This study established a prediction model of dPET/CT metabolic parameters for LNM for the first time. Although the difference in Ki between the LNM group and the non-LNM group was statistically significant in the univariate analysis, it was not an independent predictor and may need to be further verified by expanding the sample size. Recent studies have confirmed the potential of dynamic TAC to distinguish between lung cancer and benign lung lesions (19). Therefore, we further incorporated TAC features into our prediction model and found that the slope_10–30_ in the PET/CT model can effectively predict LNM. Slope_10–30_ accounts for the information of the mid-phase retention. The radiotracer activity changes within the first 10 minutes post-injection are more susceptible to the influence of blood perfusion. Conversely, the changes in radiotracer activity tend to stabilize after 30 minutes post-injection. The Slope_10–30_ can be derived from scans conducted between 10 and 30 minutes post-injection, facilitating a protocol that substantially decreases the required scan duration. This reduction in scan time can enhance scanning efficiency and lower the overall costs associated with large-scale studies. Additionally, a shorter scan duration offers the added benefit of minimizing the likelihood of motion artifacts, thereby potentially improving image quality. Therefore, we designed the feature of the TAC (Slope_10-30)_ to better capture the metabolic uptake characteristics of the target lesion.

According to reports, CA125 was significantly correlated with TNM staging of NSCLC and could provide additional prognostic information (27–29). Although its specificity in the diagnosis of lung cancer was low (25), CA125 was the only independent predictor of LNM in the clinical model of this study (OR = 1.032, *P* = 0.005). It is exciting that the predictive performance of the composite model (AUC = 0.867, *P* < 0.001) combining clinical and metabolic parameters was better than that of a single PET/CT model (AUC = 0.822, *P* < 0.001) or a clinical model (AUC = 0.792, *P* < 0.001), indicated that multidimensional integration can compensate for the limitations of a single parameter and improve diagnostic accuracy.

In addition, our study had several limitations. Firstly, this study is a retrospective study, and there may be selection bias in the small number of cases included, which may affect the extrapolation of results. Secondly, 11 of the 60 patients with non-LNM included in this study were confirmed by EBUS, which may affect the accuracy of the study results. In the future, it is necessary to expand the sample size confirmed by surgery to further verify the results. Another limitation is that our study assumes K4 = 0, but the reversibility of FDG uptake has been confirmed (30, 31), so there is a certain gap between our results and the real results, which affects the accuracy of absolute quantification. And our study does not consider the difference in uptake between blood and plasma is a limitation of the study, this omission can introduce bias in kinetic parameter estimation, particularly for K1 and Ki. In addition, our research model has completed cross validation, but there is the risk of overfitting. Last, dynamic scanning was time-consuming and required professional post-processing, which limited its widespread clinical application. Finally, the lack of external validation in the study required validation of the model’s generalization ability in an independent queue. In the future, it is still necessary to expand the sample size and enhance model stability through prospective multi-center studies.

Although there are some limitations in our research, there are also some significant advantages. First, the dPET data rather than relying solely on static SUVs, then the integration of kinetic, TAC-derived, and volumetric parameters, and finally the combination of imaging biomarkers with serum tumor markers (CA125), which improves predictive performance.

## 5 Conclusion

In conclusion, our study confirms that PET/CT parameters TLG and slope_10–30_ combined with CA125 can effectively predict LNM in NSCLC, providing a new approach for individualized staging. However, further research with larger sample sizes is needed to validate these findings.

## Data Availability

The original contributions presented in the study are included in the article/supplementary material. Further inquiries can be directed to the corresponding authors.
